# Vascular dementia: Cognitive, functional and behavioral assessment. Recommendations of the Scientific Department of Cognitive Neurology and Aging of the Brazilian Academy of Neurology. Part II

**DOI:** 10.1590/S1980-57642011DN05040004

**Published:** 2011

**Authors:** Eliasz Engelhardt, Carla Tocquer, Charles André, Denise Madeira Moreira, Ivan Hideyo Okamoto, José Luiz de Sá Cavalcanti, Working Group on Alzheimer’s Disease and Vascular Dementia of the Brazilian Academy of Neurology

**Affiliations:** 1 Full Professor (retired) – UFRJ, Coordinator of the Cognitive Neurology and Behavior Sector, INDC, CDA/IPUB, UFRJ, Rio de Janeiro RJ, Brazil; 2 Neurologist, Masters and PhD in Neuropsychology, Claude Bernard University, France; 3 Associate Professor of Neurology, Faculty of Medicine, UFRJ. Medical Director of SINAPSE Rehabilitation and Neurophysiology, Rio de Janeiro RJ, Brazil; 4 Adjunct Professor of Radiology, School of Medicine, UFRJ. Head of Radiology Sector, INDC, UFRJ, Rio de Janeiro RJ, Brazil; 5 Department of Neurology Neurosurgery, UNIFESP, Institute of Memory, UNIFESP, São Paulo SP, Brazil; 6 Adjunct Professor of Neurology, INDC, UFRJ. Cognitive Neurology and Behavior Sector, INDC, UFRJ, Rio de Janeiro RJ, Brazil

**Keywords:** recommendations, vascular dementia, neuropsychology, activities of daily living, behavioral and psychological symptoms, diagnosis disclosure

## Abstract

Vascular dementia (VaD) is the most prevalent form of secondary dementia and the second most common of all dementias. The present paper aims to define guidelines on the basic principles for treating patients with suspected VaD (and vascular cognitive impairment - no dementia) using an evidence-based approach. The material was retrieved and selected from searches of databases (Medline, Scielo, Lilacs), preferentially from the last 15 years, to propose a systematic way to assess cognition, function and behavior, and disease severity staging, with instruments adapted for our milieu, and diagnosis disclosure. The present proposal contributes to the definition of standard diagnostic criteria for VaD based on various levels of evidence. It is noteworthy that only around half of the population of patients with vascular cognitive impairment present with dementia, which calls for future proposals defining diagnostic criteria and procedures for this condition.

## Abstract (pt)

A demência vascular (DV) é a forma de demência secundária mais prevalente e a segunda entre todas as demências. O presente artigo visa estabelecer diretrizes dos princípios básicos para o atendimento de pacientes com suspeita de DV (e comprometimento cognitivo vascular - não demência), fundamentadas em evidência. O material foi obtido e selecionado a partir de busca em bases de dados (Medline, Scielo, Lilacs), preferencialmente dos últimos 15 anos, para propor a sistemática da avaliação cognitiva, funcional e comportamental, além do estadiamento da gravidade, com instrumentos adaptados para o nosso meio, e a revelação do diagnóstico. A presente proposta contribui para a definição dos padrões de diagnóstico da DV através de evidência comprovada em vários níveis. É ressaltado que apenas cerca da metade da população dos pacientes com comprometimento cognitivo vascular apresenta quadro de demência, o que torna necessária, futuramente, uma proposta visando o estabelecimento de critérios e elaboração diagnóstica dessa condição.

## Introduction

Vascular dementia (VaD) is characterized by cognitive impairment, functional decline, behavioral disorders and neurological symptoms secondary to cerebrovascular disease (CVD). Vascular cognitive impairment (VCI) includes from very mild forms of impairment (VCI no dementia [CIND] and vascular mild cognitive impairment [VMCI]) to more severe forms, including VaD,^1-5^ thus constituting a VCI/VaD spectrum. CVD can manifest associated to AD, constituting mixed forms such as AD+CVD and MD.^6-9^ Pure forms of VCI/VaD associated to AD constitute vascular cognitive disorder (VCD),^10^ a concept later incorporated into VCI.^11^ VaD (and likewise CIND) is a clinically and anatomically heterogeneous condition with the underlying physiopathologic features outlined.^12^

The goal of the working group involved in the module "Vascular Dementia: diagnostic criteria and supplementary exams" was to put forward basic guidelines based on evidence for diagnosing VaD. This is the first task of this kind undertaken on VaD in our milieu having led to a preliminary publication of a version of these guidelines.^12^

The previously published version was revised and split into two parts:

- diagnostic criteria and supplementary exams (part I).
- cognitive, functional and behavioral assessment (part II).

This second section of the diagnostic module for VaD defines the instruments for cognitive, functional and behavioral assessment, with special emphasis on versions validated for use in Brazil. The issue concerning disclosure of the diagnosis also addressed.

## Methods

The guidelines (recommendations and suggestions) were based on publications retrieved from electronic databases (Medline, Scielo, Lilacs) and encompassed scientific articles, systematic reviews, meta-analyses, largely published within the last 15 years, or earlier when pertinent. Consensus and Studies on the theme or related subjects were also examined.^13-22^

### Classification of evidence and levels of recommendation

The scientific evidence for diagnostic assessment was evaluated according to pre-established levels of certainty (Classes I, II, III and IV) and recommendations were graded according to strength of evidence (Level A [standard], B [normal], C [reduced clinical certainty], and additionally Practical option [questionable clinical certainty] and "*Good Practice Point*" [base on the experience and consensus of the task group]), in accordance with the definitions produced based on EFNS and AAN guidance.^15,23^

These guidelines may not be applicable under some circumstances and decisions on whether to apply recommendations must be taken in light of the individual clinical presentation of the case and of the resources available.^23^ Classification of evidence and recommendation levels has been described in detail.^12^

### Neuropsychological assessment

**Diagnostic test selection** - Given the variety of lesions possible in VaD, the disease can lead to a broad range of cognitive changes. With regard to the VCI/VaD spectrum, impairment can be relatively mild to more severe, with the added possibility of association of CVD with AD. The pattern of cognitive alterations varies and neuropsychological protocols must offer sensitivity to detect a wide range of domains, particularly executive function. The tests selected must meet the criteria of frequency and of validity, be freely available, well known and sensitive for detecting cognitive decline. The protocols must be broad, easy to administer and relatively brief.^14,20,24-27^ Tests offer qualitative and quantitative data, and the latter must be endowed with normative values for each test, including differentiated values for age and schooling variables, and ideally specific to the Brazilian milieu. Quantification must be expressed as mean and standard deviation (m±sd) and/or in percentiles. The values clearly within normal range are between m±1sd [16^th^ to 84^th^ percentile]. A score of between 1 and 2 sd below the mean can be considered borderline abnormal (mild cognitive impairment), scores between 2 and 3 sd below the mean as abnormal, and 3 sd below the mean as clear-cut abnormal. However, values of m-2 sd [percentile of 2] may potentially contain individuals within normal range, albeit representing a minority population. The choice of a strict cut-off point may not always be the most fitting or desirable approach for clinicians due to the risk of excluding individuals with very mild dementia, where higher cut-off points can instead be adopted for better detection of possible dementia cases. Quantitative and qualitative data from neuropsychological assessments should be taken into account, together with information from other instruments, as well as the anamnesis, in reaching a clinical decision on the definition of the condition.^28,30^

Considering the different fields of application of neuropsychological assessment (clinical practice or clinical research), protocols with specific scope and duration are required. For instance, the purpose of a screening protocol is for primary care application in the doctor's office or patient's domicile (or at bedside). A longer protocol is best however, for more in-depth studies in a clinical research setting, or a briefer version for use in clinical practice.

Neuropsychological assessment is important for several reasons:

- The diagnosis of dementia depends on evidence of cognitive impairment, involving memory, language, praxis, gnosia and executive function (required in diagnostic criteria, e.g. DSM-IV);
- Medical specialists increasingly see patients in initial phases of the disease, in whom the early detection of specific disorders is paramount, preferably before symptoms evolve to dementia thresholds.

Neuropsychological assessment must be performed by an experienced neuropsychologist in order to enable the identification of mild cognitive impairment or mild or moderate dementia. The knowledge held by the physician, besides aiding diagnosis, also comes to bear in patient management.^14^

The tests for assessing the main cognitive domains are outlined below.

### Global cognitive function

The Mini-Mental State Examination (MMSE)^31^ constitutes a screening instrument (often applied at first interview). The exam is useful as a global cognitive assessment and to help detect cognitive impairment (Class I). The sensitivity of the instrument increases when a longitudinal decline in score is observed.^21^ The MMSE has undergone a number of validations in our milieu, controlling for schooling and age, with results of several authors available in the literature.^21^

Broader and more in-depth global cognitive assessment can be achieved using the CAMCOG, part of the CAMDEX (Cambridge Examination for Mental Disorders of the Elderly).^33^ The instrument can be applied in part (subscales) or whole, depending on the protocol followed. The CAMCOG-R, a revised version of the instrument,^34^ contains an executive function subscale, although is considered lacking in efficacy to reach a specific diagnosis.^35^ The CAMCOG has proven suitable in diagnostic assessments for longitudinal follow-up of patients with different types of dementia including VaD,^36-38^ registering decline of 12-14 points over a one year period in the absence of treatment.^39^ The instrument has been translated and validated for use in Brazil, including global normative studies and subscales, with differentiated cut-off scores controlling for schooling and age.^40-43^ An assessment of a VaD patient sample showed significantly lower global and subscale scores on the CAMCOG compared to normal controls (Class I).^44^

The CDR scale (Clinical Dementia Rating scale) is an instrument for determining severity and staging which is also useful for global assessment (Class IV).^45^

### Orientation

This can be assessed using the appropriate subscales from the MMSE,^3^ previously translated and validated by a number of investigators^32^ for use in our milieu, or by applying the subscale from the CAMCOG. Some studies have reported better orientation in VaD compared with AD, while others failed to observe significant differences.^46^ In our milieu, lower scores on orientation were found in VaD patients (CAMCOG) compared with normal controls.^44^

### Attention

Attention can be assessed using the digit span sub-test (WAIS-III),^47^ which entails repeating an increasingly longer sequence of digits in forward and reverse order. The WAIS-III was translated and adapted for our milieu, with the inclusion of normative values,^48,49^ and a comprehensive study performed on the digit span subtest.^50^ The results of several studies showed no significant difference between VaD and AD on this subtest.^46^ The CAMCOG contains an attention subscale and studies in Brazil have shown significantly lower scores in VaD patients compared with normal controls.^44^ The Trail Making Test can also yield information on attention, where the A form assesses focused attention and the B form provides data on divided attention (also see "Executive function").^51^

### Memory

All diagnostic criteria include some form of memory impairment. Both episodic (recent) and semantic (remote) modalities should be assessed. Episodic memory reflects initial compromise in AD^52^ and includes some characteristics specific to VaD (particularly subcortical) compared with AD. Spontaneous evocation of verbal material proved superior while no relevant differences were found for non-verbal content. Evocation can be improved by using cues. Recognition on re-presentation was also greater in VaD (Class III).^46,54,55^ Few studies have been conducted on semantic VaD and results available are controversial showing the same, greater or less impairment compared to AD.^53^ The two modalities of memory can be assessed using memory subscales from the CAMCOG.^41^ A Brazilian study has shown significantly lower scores on the memory subscale (total) in VaD compared to normal controls, with worse spontaneous evocation and partial improvement with cues. In vascular CIND, spontaneous evocation was found to be better compared with VaD and the benefits from use of cues was similar to that seen in normal controls.^44^ Learning and memory can be assessed using the word list from CERAD^55^ which has been translated and validated for use in Brazil.^56,57^ Patients with VaD have clearly better verbal learning and memory performance based on word list tasks compared to subjects with AD (confirmed in 61% of studies).^46^

### Executive function

Executive dysfunction (dysexecutive syndrome)(ED) has become a prominent and essential feature for diagnosis of VaD, especially the subcortical subtype, making a formal assessment of this domain necessary.^58-60^ Studies focusing on ED using specific instruments have shown more severely compromised performance in VaD (and MD) compared to AD (Class III).^46,53,61,62^ The assessment can be performed using Verbal Fluency tests (semantic and phonemic),^63^ the Trail Making Test,^64,65^ variants of the Clock Drawing Test,^63,66^ the abstract thinking subtest (CAMCOG), as well as assessment of working memory. The need to use several tests to assess ED is owing to the extent of the condition and the multiple frontal functions involved,^67^ and allows for better detection of one given performance over another, in light of the clinical heterogeneity of VaD.

**Verbal fluency** - The semantic verbal fluency test (animals category)(SVF)is one of the most commonly used tests. The test is subject to lexical knowledge and semantic memory and appears to depend on interaction of frontal and temporal areas.^68^ The phonemic verbal fluency test (PVF) is also widely used, representing a sensitive task for assessing frontal function (especially the left prefrontal area).^63^ The SVF has discriminative value in differentiating cognitive impairment and dementia from that found in normal aging, and similarly in VaD and AD.^54,64^ A study comparing SVF (animals category) and PVF (letter F) showed that patients with AD and CIND had poorer scores than individuals with VaD (and vascular CIND). The SVF test was superior for discriminating all patients compared with normal controls.^69^ The SVF has been validated for use in Brazil taking into account schooling and age with, akin to the PVF, differentiated cut-off scores for elderly age groups and educational level.^71^ A study in VaD patients revealed significantly poorer performance on the SVF and PVF compared to normal controls and AD patients.^72^ Another study investigating VaD and control groups showed significantly lower scores on the SVF in VaD subjects^44^ and also in a sample of VaD and MD patients.^73^

**Trail Making Test** - This test (TMT) is intended to reflect broad and complex variety in cognitive processes (e.g. attention, visuomotor sequencing and alternation, cognitive flexibility, psychomotor speed).^63,74,75^ Performance depends on diverse anatomic structures, including the medial part of the temporal lobe, with atrophy and extent of white matter lesion strongly influencing task completion time.^76^ The TMT has two forms (A and B). The test is timed, yielding data on processing speed (visuomotor) (TMT-A). In addition, the test provides a measure of cognitive flexibility (TMT-B), particularly regarding the relationship B/A >3 (Class II).^76,77^ TMT has been shown to differentiate patients with cerebral small vessel disease from healthy controls.^27^ The test is commonly used in our milieu, despite not being validated, where one study correlated schooling and age to performance and also reported normative data.^51^ Significantly higher scores have been registered on the TMT-A and TMT-B in VaD patients^44^ and subjects with VaD and MD,^73^ versus normal controls.

**Clock Drawing Test** - This test (CDT)(and its variants) has been the focus of numerous publications and a number of scoring systems (scales ranging from 3 to 10 points). Besides a cognitive screening instrument for dementia, the tool is used to assess executive function and visuoconstructive ability.^78^ The CAMCOG incorporates a version of the CDT (scoring scale 0-3).^38^ A study conducted using this variant has demonstrated that patients with VaD have poorer performance and proven able to correctly classify 65.9% of a sample into AD and VaD groups.^79^ The CDT in our setting is influenced by age and schooling, and appears unsuitable for screening dementia in elderly persons with ≤4 years of schooling.^80^

A variant of the CDT called the "Clock Drawing Executive Task"(CLOX), is an instrument comprising two parts (CLOX1 [drawing by instruction] and CLOX2 [drawing by copying]). Better performance on the CLOX2 suggests executive dysfunction.^66^ Concerning VaD patients, the CLOX was applied to a sample of patients with AD and also to a mixed vascular group (MVG=AD+CVD and VaD) and compared to a control group. The scores obtained differed significantly, with worse performance by the MVG group, particularly on the CLOX1, allowing differentiation among the samples.^81^ Application of the instrument in our milieu among patients with mild forms of subcortical VaD and AD in comparison to normal controls produced differentiated scores on CLOX1/CLOX2, discriminating VaD from AD patients (Class II).^72^

**Abstract thinking** - This is the ability to draw similarities (e.g. among objects) and can be tested using the subscale on the CAMCOG. A study in VaD patients (and vascular CIND) versus normal controls showed statistically lower scores in the former on this subscale.^44^

**Working memory** - This can be assessed in the domain of the ED.^82^ Results vary in VaD (compared to AD) depending on the subtype considered.^46^ The verbal modality can be assessed by the digit span subtest, particularly the inverse order (WAIS-III).

### Instrumental functions

Instrumental functions, such as oral (comprehension and expression) and written (reading and writing) language, calculus, praxis and gnosia, including visuospatial and visuoconstructive abilities, can also be affected to varying degrees, being particularly impaired with cortical lesions (infarcts) in subtypes of VaD. Comparison with AD reveals variable differences.^46,56^ Language (plus calculus) can be assessed by means of the subscales from the CAMCOG. A brief version of the Boston Naming Test (CERAD),^55^ translated and validated for Brazilians,^56^ may also be applied. The SVF animals category serves as a less structured lexical retrieval task (besides testing executive function as indicated) and has long been in use, offering some utility in discriminating cognitive impairment due to normal aging from impairment due to dementia, such as VaD and AD.^54,69^ Tests assessing aphasia and correlated functions tend to be longer and more time-consuming to apply, only being used under special circumstances. The domains praxis and gnosia (including visuospatial and visuoconstructive abilities) also have dedicated subscales in the CAMCOG (drawing items). A study conducted in Brazil found significantly worse scores on language subscales (total), calculus, praxis, gnosia, SVF and naming, in VaD compared with normal controls.^44^

**Recommendations** - Neuropsychological assessment (cognitive) is essential in diagnosing (and managing) VaD, and should be carried out in all patients. In addition to global cognitive assessment, more in-depth tests should be applied for main cognitive domains, including memory, instrumental and executive functions, considering qualitative and quantitative aspects (*Level A*).

### Assessment of activities of daily living

The decline in everyday functional skills (activities of daily living - ADL) is an important component of the dementia syndrome, and its assessment is an integral part of the diagnostic process. Different scales are used to objectively measure these abilities based on interviews with the caregiver of the patient. The ADL assessed include basic and instrumental type activities. Executive dysfunction has been linked to worse performance on functional measures as well as to worse short-term outcomes.^62^ A frequently used scale is the Functional Activities Questionnaire (FAQ) which assesses functional capacity based on degree of independence for performing instrumental activities of daily living and social cognitive functions.^83^ The FAQ comprises 10 items scored from 0 to 3, with a maximum total score of 30 points. The higher the score on the questionnaire, the greater the degree of dependence of the patient with scores ≥6 reflecting functional loss.^84^ The FAQ has been translated and applied in Brazil by several groups.^85^ One such study found that VaD patients had significantly higher scores on the FAQ compared to normal controls.^44^

**Recommendations** - Compromised activities of daily living and cognitive impairment is an essential part of dementia criteria and should therefore be investigated in diagnostic assessments (*Good Practice Point*).

### Assessment of behavioral and psychological symptoms

Behavioral and Psychological Symptoms of Dementia (BPSD)("neuropsychiatric disorders") are frequent manifestations in dementia. These symptoms contribute to patient suffering and caregiver burden, constituting the major factor leading to the prescription of psychotropic agents and to institutionalization.^86,87^ The time course of these symptoms can vary, occurring at different points during disease evolution. BPSD can be exacerbated, or caused, by somatic comorbidities.^88^ A number of instruments are available for assessment, with the majority relying on informant reports. The Neuropsychiatric Inventory (NPI) is one such scale in frequent use which assesses many symptoms (delusions, hallucinations, psychomotor agitation, depression, anxiety, euphoria, apathy, disinhibition, irritability, aberrant motor behavior, night-time behavior and eating changes), scored according to presence, frequency, severity and caregiver impact.^89^ A translated and validated version of the NPI is available for application in Brazil.^90^

The NPI has been used for assessing VaD in two key studies. The first of these studies showed that the most frequent symptoms of VaD were depression, agitation/aggression and apathy, followed by psychosis, irritability and anxiety.^91^ Another study comparing findings in VaD in small vessel and large vessel diseases found apathy to be the most prevalent symptom, followed by depression, irritability and agitation/aggression. Patients with small vessel VaD showed greater apathy, aberrant motor behavior and hallucinations whereas large vessel VaD patients exhibited more severe agitation/aggression and euphoria.^92^ A Brazilian study comparing VaD patient found significantly higher scores on the NPI in VaD (and also in Vascular CIND) compared to healthy controls.^44^ Stratification of the NPI results showed predominance of apathy, depression and anxiety, followed by irritability and agitation, sleep disorders, psychosis, and other manifestations to a lesser degree, in a sample of patients with VaD and MD (Class II).^73^

The assessment of the presence of depression is also advisable, with the Cornell scale (CSDD) frequently used for this purpose. A score ≥8 is suggestive of significant depressive symptomatology.^93^ The scale has been translated and validated for use in Brazilian subjects.^94^ Depression was assessed in Brazil using the Cornell scale and revealed significantly higher scores in VaD (and also in vascular CIND), compared to normal controls.^44^

**Recommendations** - Assessment of BPSD is essential in diagnosing and managing VaD and should be carried out in all patients (*Level A*). A comorbidity of some kind should always be considered as a possible cause (*Level C*). Symptoms must be actively inquired about in both patients and active caregivers, an using appropriate scale or scales (*Good Practice Point*).

### Dementia staging

The CDR scale (Clinical Dementia Rating scale) is a widely used qualitative instrument for determining severity and staging of dementia originally developed for AD.^95,96^ The scale has been translated and validated for use in Brazil having been applied to a sample of AD and VaD patients (approximate 1:1 ratio). The CDR identified and stratified 207 dementia patients into 3 stages - CDR1 (34%), CDR2 (42%) and CDR3 (22%). The instrument had 86% sensitivity and 100% specificity, for patients with dementia and healthy elderly (Class IV). Notably, no influence from schooling was observed on the patients classified into different CDR categories, suggesting a lesser impact of this parameter on this instrument.^97,98^ The validity of the scale exclusively for VaD has yet to be established.

**Recommendations -** Clinical staging of dementia should be systematically assessed in a serial manner in order to determine the current severity and evolution of the disease (*Good Practice Point*).

### Proposed clinical assessment protocols

Protocols of different lengths were proposed for VCI.^14,27^ Table 1 shows a proposed brief screening protocol (Protocol A) and Table 2 shows proposed tests for a longer protocol (Protocol B). In a bid to maximize information obtained, widely used conventional tests were selected, preferably translated and validated for use in Brazil.

**Table 1:** Protocol A. Proposal for screening (see text for original references, translations and validations).

Tests	MMSE (global)
Verbal Fluency (animals)	CDT (CAMCOG)

MMSE: Mini-Mental State Examination; CDT: Clock Drawing Test.

**Table 2:** Protocol B (see text for original references, translations and validations).

Tests
Global
MMSE (global) (screening), CAMCOG (global)

Orientation
Time and space (MMSE or CAMCOG) [10 points][120-129]

Attention
Attention subscales (CAMCOG) [7 points] [159-160]
Digit span [do and io]* (WAIS-III)
Trail Making Test (TMT) [forms A and B]

Memory
Memory subscale (CAMCOG) [27 points] [146-157, 178]
Words list (CERAD)**

Executive function
Semantic Verbal Fluency (animals)
Phonemic verbal fluency (F-A-S)**
Abstraction (CAMCOG) [8 points] [179-182]
Trail Making Test (TMT) [forms A and B]
CDT (CAMCOG)
CLOX (1 and 2)
Working memory (digit span - io}(WAIS-III)**

Language
Language subscale (CAMCOG) [30 points]
[items 130-136, 138-144, 162-163, 171]
Language subscale - naming (CAMCOG) [8 points] [138]
Verbal Fluency (animals)
Naming (Abbreviated CERAD or Boston)**

Praxis and visuoconstructive abilities
Praxis subscale (CAMCOG)[12 points]
[164-167, 170, 172-174]
Gnosia and visuospatial abilities
Gnosia subscale (CAMCOG) [11 points] [175, 183-185]
Function (ADL) Functional Activities Questionnaire (FAQ)

Behavioral and Psychological symptoms
Neuropsychiatric Inventory (NPI)
Depression scale (CSDD - Cornell scale)

Staging
CDR (Clinical Dementia Rating Scale)

* do: direct order; io: inverse order,

** optional.

### Diagnosis disclosure

Scant knowledge is available on the issue of diagnosis disclosure in terms of physicians' attitudes and reaction of patients and their family members. Studies on the subject focus on the problem in connection with AD while the majority of research was conducted internationally (EU, UK and USA). The guidelines tend to vary and often differ according to the type of healthcare professional involved (generalists, specialists etc.). Clinical complexity and aspects relating to cultural diversity must be considered.^99-102^ An extensive review of the literature on existing evidence regarding disclosure of dementia diagnosis has shown inconsistent and limited results, where the perspective of the impaired individual is generally overlooked. The state of knowledge on the issue has led to disparities among the diverse proposed guidelines currently proposed.^103^ Disclosure of the diagnosis can be considered a basic intervention in dementia management and should be done without causing undue stress for patient and caregiver, and must aid orientation. The practice of disclosure should be carefully planned and executed.^104^

No specific studies on diagnosis disclosure for VaD (or vascular CIND) were found. Considering VaD at mild stages (and particularly in the case of vascular CIND), disclosure can be important for encouraging adherence to treatment thereby preventing or attenuating progression to more severe stages. However, disclosure must be done cautiously, especially in older patients, given the possibility of associated neurodegenerative processes. It is important to reiterate that these associations, vascular and neurodegenerative lesions, tend to have a more marked clinical manifestation^105^ and that controlling vascular-related factors that can be treated preventively is favorable for both conditions and beneficial to the patient.

**Recommendations** - Disclosure of the diagnosis, when informed, must be done cautiously taking into account psychological and cultural characteristics of the patient, and must be accompanied by information on the possible repercussions in terms of potential disease progression. Concerning VaD (principally vascular CIND), explaining that preventive measures may lead to a more favorable prognosis can result in improved adherence of patients to such actions, even when considering possible association with the neurodegenerative process (*Good Practice Point*).

## Conclusion

The assessment procedures for diagnosing VaD require multi-disciplinary interaction toward reaching a diagnosis. This part of the proposal addressed the host of instruments for performing cognitive, functional and behavioral assessment, classified according to proven evidence at several levels, used for diagnosing VaD, and also expounds on diagnosis disclosure.

It should be highlighted that only around half of the population of patients with VCI/VaD present with dementia. Our group envisages that the proposed guidelines can be further refined to enable more accurate diagnosis of this condition and that part of the spectrum of CIND and vascular MCI can be extended with the defining of suitable criteria for diagnosis.

## GROUP RECOMMENDATIONS IN ALZHEIMER'S DISEASE AND VASCULAR DEMENTIA OF THE BRAZILIAN ACADEMY OF NEUROLOGY

**Amauri B. da Silva** [UNINEURO, Recife (PE)]; **Ana Cláudia Ferraz** [Serviço de Neurologia do Hospital Santa Marcelina (SP)]; **Analuiza Camozzato de Pádua** [Universidade Federal de Ciências da Saúde de Porto Alegre (UFCSPA); Hospital de Clínicas de Porto Alegre (UFRGS) (RS)]; **Antonio Lúcio Teixeira** [Departamento de Clínica Médica, Faculdade de Medicina da Universidade Federal de Minas Gerais, Belo Horizonte (MG)]; **Ayrton Roberto Massaro** [Instituto de Reabilitação Lucy Montoro (SP)]; **Benito Pereira Damasceno** [Departamento de Neurologia da Universidade Estadual de Campinas (SP)]; **Carlos Alberto Buchpiguel** [Departamento de Radiologia, Faculdade de Medicina da Universidade de São Paulo (SP)]; **Cássio Machado C. Bottino** [Programa Terceira Idade, Instituto de Psiquiatria do Hospital das Clínicas da Faculdade de Medicina da Universidade de São Paulo (FMUSP) (SP)]; **Cláudia C. Godinho** [Serviço de Neurologia do Hospital de Clínicas de Porto Alegre, Universidade Federal do Rio Grande do Sul (RS)]; **Cláudia Sellitto Porto** [Grupo de Neurologia Cognitiva e do Comportamento da Faculdade de Medicina da USP (SP)]; **Delson José da Silva** [Núcleo de Neurociências do Hospital das Clínicas da Universidade Federal de Goiás (UFG); Instituto Integrado de Neurociências (IINEURO), Goiânia (GO)]; **Elza Dias-Tosta** [Presidente da Academia Brasileira de Neurologia, Hospital de Base do Distrito Federal (DF)]; **Emílio Herrera Junior** [Departamento de Medicina Interna, Faculdade de Medicina de Catanduva (SP)]; **Francisco de Assis Carvalho do Vale** [Universidade Federal de São Carlos (UFSCar), Departamento de Medicina (DMed) (SP)]; **Gabriel R. de Freitas** [Instituto D'or de Pesquisa e Ensino; Universidade Federal Fluminense (RJ)]; **Hae Won Lee** [Instituto de Radiologia, Hospital das Clínicas da Faculdade de Medicina da Universidade de São Paulo e Hospital Sírio-Libanês (SP)]; **Jerusa Smid** [Grupo de Neurologia Cognitiva e do Comportamento do Hospital das Clínicas da Faculdade de Medicina da Universidade de São Paulo (FMUSP) (SP)]; **João Carlos Barbosa Machado** [Aurus IEPE - Instituto de Ensino e Pesquisa do Envelhecimento de Belo Horizonte; Faculdade de Ciências Médicas de Minas Gerais (FCMMG), Serviço de Medicina Geriátrica do Hospital Mater Dei (MG)]; **José Antonio Livramento** [Laboratório de Investigação Médica (LIM) 15, Faculdade de Medicina da Universidade de São Paulo (SP)]; **Letícia Lessa Mansur** [Grupo de Neurologia Cognitiva e do Comportamento do Departamento de Neurologia da FMUSP; Departamento de Fisioterapia, Fonoaudiologia e Terapia Ocupacional da Faculdade de Medicina da USP (SP)]; **Márcia Lorena Fagundes Chaves** [Serviço de Neurologia do Hospital de Clínicas de Porto Alegre, Universidade Federal do Rio Grande do Sul (RS)]; **Márcia Radanovic** [Laboratório de Neurociências - LIM27, Departamento e Instituto de Psiquiatria da Faculdade de Medicina da Universidade de São Paulo (FMUSP) (SP)]; **Márcio Luiz Figueredo Balthazar** [Universidade Estadual de Campinas (UNICAMP), Faculdade de Ciências Médicas (FCM), Departamento de Neurologia (SP)]; **Maria Teresa Carthery-Goulart** [Grupo de Neurologia Cognitiva e do Comportamento do Departamento de Neurologia da Faculdade de Medicina da USP; Centro de Matemática, Computação e Cognição, Universidade Federal do ABC (SP)]; **Mônica S. Yassuda** [Grupo de Neurologia Cognitiva e do Comportamento do Departamento de Neurologia da Faculdade de Medicina da USP; Departamento de Gerontologia, Escola de Artes, Ciências e Humanidades da USP (EACH/USP Leste) (SP)]; **Nasser Allam** [Universidade de Brasília (UnB), Laboratório de Neurociências e Comportamento, Brasília (DF)]; **Norberto Anizio Ferreira Frota** [Universidade de Fortaleza (UNIFOR), Serviço de Neurologia do Hospital Geral de Fortaleza (HGF) (CE)]; **Orestes Forlenza** [Laboratório de Neurociências - LIM27, Departamento e Instituto de Psiquiatria da Faculdade de Medicina da Universidade de São Paulo (FMUSP) (SP)]; **Paulo Caramelli** [Departamento de Clínica Médica, Faculdade de Medicina da Universidade Federal de Minas Gerais, Belo Horizonte (MG)]; **Paulo Henrique Ferreira Bertolucci ** [Universidade Federal de São Paulo (UNIFESP), Setor de Neurologia do Comportamento - Escola Paulista de Medicina, São Paulo (SP)]; **Regina Miksian Magaldi** [Serviço de Geriatria do Hospital das Clínicas da FMUSP, Centro de Referencia em Distúrbios Cognitivos (CEREDIC) da FMUSP (SP)]; **Renata Areza-Fegyveres** [Grupo de Neurologia Cognitiva e do Comportamento do Hospital das Clínicas da Faculdade de Medicina da Universidade de São Paulo (FMUSP) (SP)]; **Renato Anghinah** [Grupo de Neurologia Cognitiva e do Comportamento do Hospital das Clínicas da Faculdade de Medicina da Universidade de São Paulo (FMUSP); Centro de Referência em Distúrbios Cognitivos (CEREDIC) da FMUSP (SP)]; **Ricardo Nitrini** [Grupo de Neurologia Cognitiva e do Comportamento do Hospital das Clínicas da Faculdade de Medicina da Universidade de São Paulo (FMUSP); Centro de Referência em Distúrbios Cognitivos (CEREDIC) da FMUSP (SP)]; **Rodrigo Rizek Schultz** [Setor de Neurologia do Comportamento do Departamento de Neurologia e Neurocirurgia da Universidade Federal de São Paulo, Núcleo de Envelhecimento Cerebral (NUDEC) - Instituto da Memória (UNIFESP) (SP)]; **Rogério Beato** [Grupo de Pesquisa em Neurologia Cognitiva e do Comportamento, Departamento de Medicina Interna, Faculdade de Medicina, UFMG (MG)]; **Sonia Maria Dozzi Brucki** [Grupo de Neurologia Cognitiva e do Comportamento da Faculdade de Medicina da Universidade de São Paulo; Centro de Referência em Distúrbios Cognitivos (CEREDIC) da FMUSP; Hospital Santa Marcelina (SP)]; **Tânia Novaretti** [Faculdade de Filosofia e Ciências, Campus de Marília, da Universidade Estadual Paulista (UNESP) (SP)]; **Valéria Santoro Bahia** [Grupo de Neurologia Cognitiva e do Comportamento do Hospital das Clínicas da Faculdade de Medicina da Universidade de São Paulo (FMUSP) (SP)]; **Ylmar Corrêa Neto** [Universidade Federal de Santa Catarina (UFSC), Departamento de Clínica Médica, Florianópolis (SC)].
